# A modelling analysis of the effectiveness of second wave COVID-19 response strategies in Australia

**DOI:** 10.1038/s41598-021-91418-6

**Published:** 2021-06-07

**Authors:** George J. Milne, Simon Xie, Dana Poklepovich, Dan O’Halloran, Matthew Yap, David Whyatt

**Affiliations:** 1 Department of Computer Science and Software Engineering, University of Western Australia, Perth, Australia; 2 School of Medicine, University of Western Australia, Perth, Australia; 3 Department of Health, Queensland Government, Brisbane, Australia

**Keywords:** Computational biology and bioinformatics, Diseases, Health care

## Abstract

There is a significant challenge in responding to second waves of COVID-19 cases, with governments being hesitant in introducing hard lockdown measures given the resulting economic impact. In addition, rising case numbers reflect an increase in coronavirus transmission some time previously, so timing of response measures is highly important. Australia experienced a second wave from June 2020 onwards, confined to greater Melbourne, with initial social distancing measures failing to reduce rapidly increasing case numbers. We conducted a detailed analysis of this outbreak, together with an evaluation of the effectiveness of alternative response strategies, to provide guidance to countries experiencing second waves of SARS-Cov-2 transmission. An individual-based transmission model was used to (1) describe a second-wave COVID-19 epidemic in Australia; (2) evaluate the impact of lockdown strategies used; and (3) evaluate effectiveness of alternative mitigation strategies. The model was calibrated using daily diagnosed case data prior to lockdown. Specific social distancing interventions were modelled by adjusting person-to-person contacts in mixing locations. Modelling earlier activation of lockdown measures are predicted to reduce total case numbers by more than 50%. Epidemic peaks and duration of the second wave were also shown to reduce. Our results suggest that activating lockdown measures when second-wave case numbers first indicated exponential growth, would have been highly effective in reducing COVID-19 cases. The model was shown to realistically predict the epidemic growth rate under the social distancing measures applied, validating the methods applied. The timing of social distancing activation is shown to be critical to their effectiveness. Data showing exponential rise in cases, doubling every 7–10 days, can be used to trigger early lockdown measures. Such measures are shown to be necessary to reduce daily and total case numbers, and the consequential health burden, so preventing health care facilities being overwhelmed. Early control of second wave resurgence potentially permits strict lockdown measures to be eased earlier.

## Introduction

An ongoing challenge faced by public health authorities during the COVID-19 pandemic is knowing when to activate social distancing strategies, the magnitude of the measures, how to safely ease the measures once case numbers reach low levels, and how best to react when a rapidly developing outbreak occurs. We report on a detailed, model-based case study into the significant COVID-19 second wave in greater Melbourne, Australia, from June 2020 onwards. This occurred as a result of ineffective management of the mandated hotel quarantine policy for returning overseas travellers, where transmission is believed to have occurred between infectious arrivals and security staff, then among the staff, and thus to their families and friends^1,2^.

The aim of this study was to understand why the second wave of SARS-CoV-2 transmission grew so rapidly; why the initial increase in social distancing response was ineffective; what responses would have been more effective; and thus the lessons learned. The insights gained are of benefit to other countries and jurisdictions in their determination of response policy.

The Australian response to COVID-19 has kept the country largely free from large-scale transmission, such as occurred in Europe, the USA and Latin America, by halting flights from China in February, and stopping inbound travel by non-Australian residents from 20th March 2020. Robust social distancing measures were also adopted country-wide on that date and further strengthened on 26th March 2020^3–5^, resulting in substantial reductions in person-to-person contact in workplaces and the community. These measures resulted in very low levels of COVID-19 virus transmission, with almost all cases arising from returning Australians infected outside the country. All arrivals were required to enter 14 days of managed hotel quarantine. Certain Australian states (*e.g.* Western Australia and Queensland) also closed their interstate borders. The initial success of Australia in halting the arrival of infectious individuals, and managing the limited number of community transmissions via testing and contact tracing, allowed all Australian states to start easing social distancing measures from May onwards, including allowing schools to reopen, as detailed in Table S1 in the Supporting Information. The total number of COVID-19 related deaths for the whole of Australia was ~ 100 up to 6th May 2020, and stayed constant at that number for the next 8 weeks. In late May, a breakdown in hotel quarantine regulations in Melbourne, State of Victoria, followed by a number of unauthorised, large-scale family gatherings, allowed the SARS-Cov-2 virus to enter the wider population in greater Melbourne, with diagnosed case numbers increasing from June onwards^6^.

**Figure 1:**
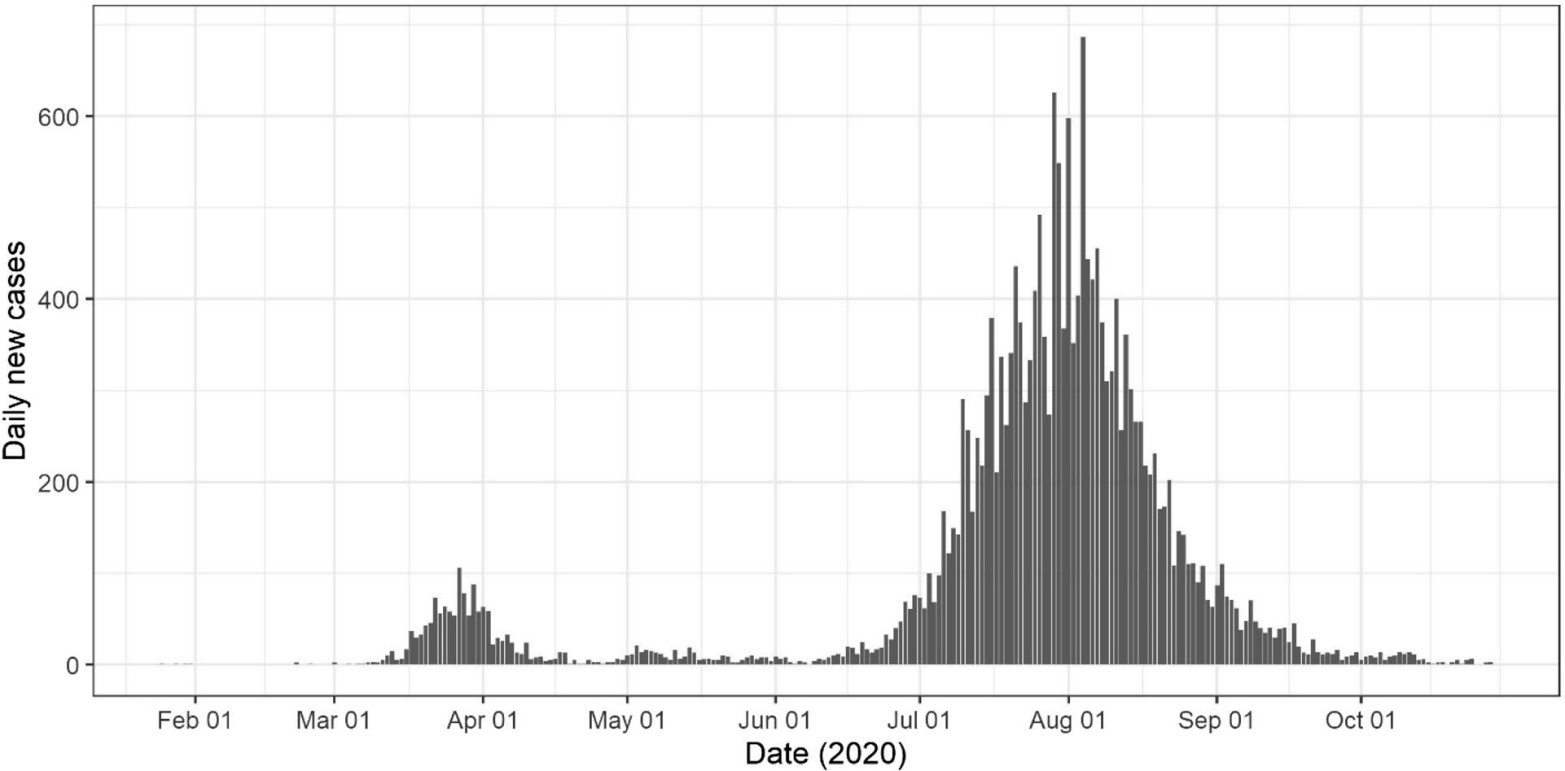
Daily COVID-19 case numbers State of Victoria, Australia. Total of 19,000 cases in greater Melbourne, and 1000 in regional Victoria, to 30th October 2020^7,8^.

Case data resulting from this second wave outbreak provided us with a unique opportunity to analyse the non-pharmaceutical (NPI) measures used, and the significance of their activation timing. This study was facilitated by the fact that Australia’s second wave was geographically contained to greater Melbourne, and not impacted by the ongoing introduction of infectious persons. This “self-contained” second wave provided us with high quality data on daily case numbers, as in Fig. 1, allowing us to evaluate second wave response measures without interference from introduced cases.

## Methods

An individual-based (*c.f.* agent-based) model capturing the demographics and movement patterns of individuals within an Australian city, together with SARS-CoV-2 virus transmission data from the early outbreak in Wuhan, China prior to social distancing activation^9^, was developed and applied. This model was used to analyse the effectiveness of a broad suite of non-pharmaceutical, social distancing interventions, by varying their strength, their time of activation, and their duration. Individual-based modelling is an appropriate method to adopt for this task. It permits the effect of four key social distancing measures to be readily captured at a high degree of detail: school closure; reduction in workplace participation; community-contact reduction; and case isolation.

Such simulation models create a “virtual world” of individuals whose population and disease biology dynamics replicate that of the real-world system in as much detail as data sources permit. These data were used to model the time-changing contact patterns for each individual, as they move between their household, school/workplace contact hubs and in the wider community.

Our study utilised an established model of the city of Newcastle (population 272,407) in the state of New South Wales, Australia. This city has population demographics which reflect that of Australia as a whole, and results were scaled to greater Melbourne, population ~ 5 million, following an established approach used previously^10,11^. This individual-based model was developed to match its real-world counterpart with respect to population, age, household structure, employment, schooling, and daily movement between these locations through the use of detailed census, workplace and mobility data from these communities, a model development and application process used previously, e.g.^12–14^. They therefore create realistic representations of the respective communities at an individual-by-individual level. The Newcastle model represents 272,407 people in the urban and suburban areas in and around Newcastle; this population is broadly representative of the Australian population as a whole in terms of age distribution.

Australian Bureau of Statistics (ABS) census data were used to capture age-specific demographics of every household in the community. Data for schools in terms of geographical location and pre-primary, primary and secondary enrolment numbers for each school were obtained from the New South Wales state government. ABS data were also used to determine household and workplace locations, and workforce sizes. These geo-located data permitted us to assign adults to workplaces, and to assign children to neighbourhood schools. Furthermore, available schools data was used to assign children from local households to age-specific classes^15–18^. All of these data components enable the models to capture the movement and contact patterns of individuals on a day-by-day basis, moving from specific households to schools and workplaces on working week days, and returning to households in the evening. The model also captured semi-random contact in the community on both weekends and, to a lesser extent, Monday through to Friday.

Model parameter settings were adopted to reflect the transmission characteristics of the COVID-19 epidemic taken from^9^. These are an incubation period averaging 6 days, from infection to symptom emergence (if any); a latent period averaging 5 days, from infection to infectious; an infectious period averaging 4.5 days, the first day being asymptomatic; and 35% of cases are asymptomatic. Each infected individual transited through the four SEIR states: susceptible; exposed but not infectious; infectious; then recovered, immune and no longer infectious. The duration within each state followed the above timelines.

Using these infection timelines the model was then calibrated to reproduce a specific reproduction number. The probability of virus transmission from an infectious individual to a susceptible individual resulting from their pairwise contact was derived for a R_0_ of 2.25, based on SARS-CoV-2 transmission characteristics from Wuhan, China obtained prior to introduction of containment measures^9,19^, see Supporting Information. The calibration process involved repeatedly adjusting the transmission probability parameter, which controls transmission between pairs of infectious and susceptible individuals who are co-located, in a series of simulation experiments. That is, running the model software with no social distancing strategies in place. For every simulation experiment the resulting infection data for each infected individual infected was used to derive a specific reproduction number. This process of adjusting the transmission probability parameter was continued until a specific parameter was found to produce a reproduction number of 2.25.

Model outputs obtained by running the simulation software produced the infection history of every individual in the community, generating the daily (and total) number of infectious individuals, and determining where and when infection occur, as in^12,20^. Modelling analyses were conducted for alternative social distancing strategies, by varying the strength of measures and their activation timing. This predicted how alternative mitigation strategies would perform, allowing us to contrast alternative mitigation strategies with those used, in terms of cases prevented.

Four social distancing measures were combined during the Melbourne COVID-19 second wave. School closure (SC); workplace non-attendance (WN); community contact reduction (CCR); and case isolation (CI). The effect of Stage 3 and Stage 4 social distancing measures on person-to-person contact patterns was estimated from Victorian state government directives, such as for age-specific school non-attendance. Workplace and community-wide contact reductions were estimated from the directives, and from observation of commuter traffic reductions. The case isolation setting allowed for a limited level of non-compliance. Details of Stage 2, Stage 3 and Stage 4 second wave response measures applied in greater Melbourne are described in the Supporting Information.

Simulation experiments were conducted by running model (simulation) software, after adjusting the strength of social distancing measures to reflect introduction of Stage3 and Stage 4 restrictions. Random seeding of infectious individuals into the model was used to capture the effect of localized high transmission events, resulting from a number of large gatherings in late June 2020^2^.

Seeding of infectious individuals into the model was stopped on 9th July 2020, thus all further infections in the model occurred as a consequence of the breakdown in hotel quarantine measures and, subsequent large family gatherings. This replicates what is known to have occurred, with genomic sequencing recently showing that over 90% of all outbreak cases were due to a breakdown in quarantine measures, and arose from transmission between infected travellers and quarantine facility staff^1,2^.

## Results

### Response timing

**Figure 2:**
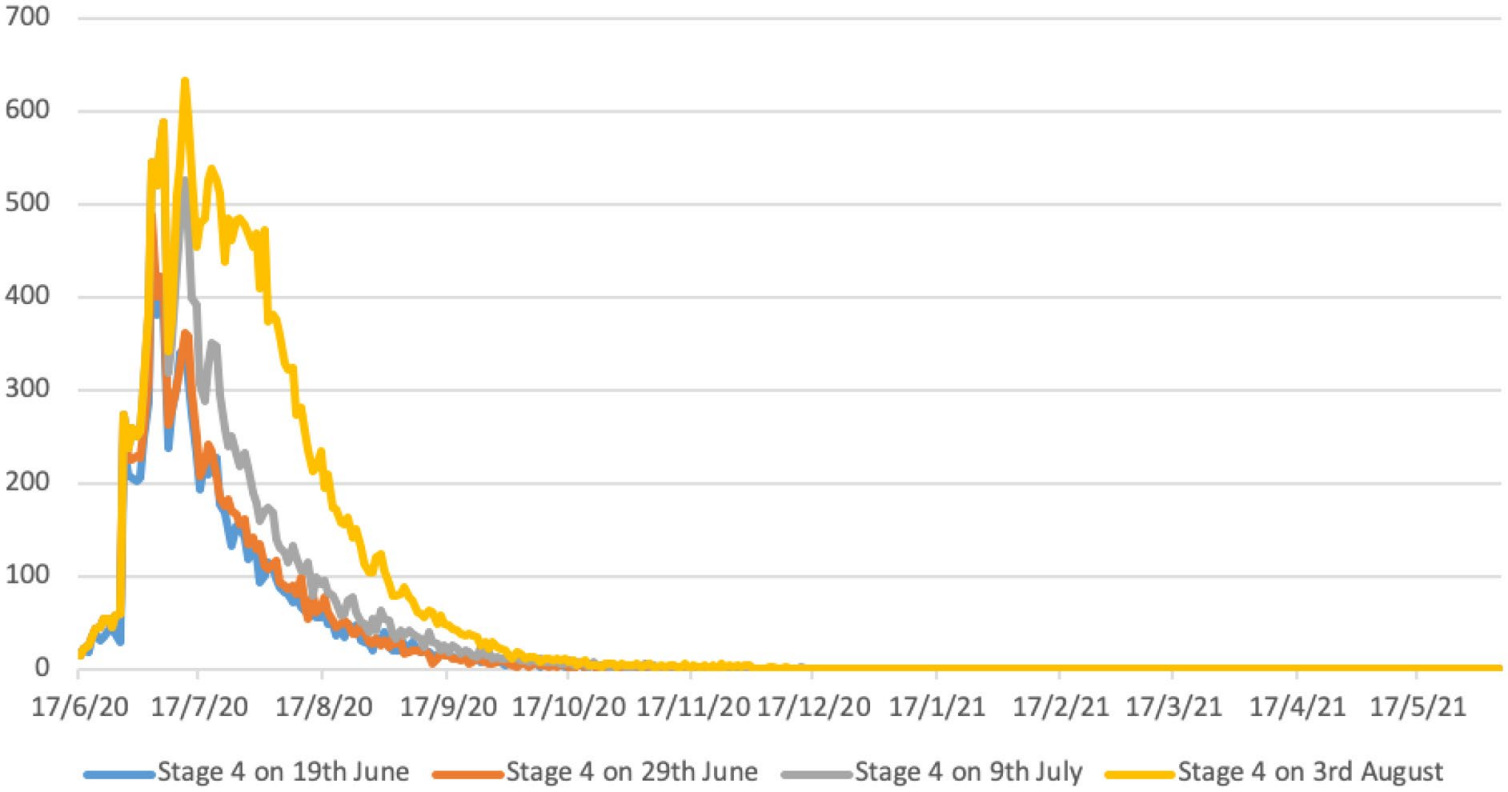
Predicted epidemic curves for earlier activation of Stage 4 lockdown measures in greater Melbourne (population 5 million). Y axis represents daily diagnosed cases. All scenarios assume schools are closed, and there is 90% case isolation for adults and 100% for children. X axis gives calendar dates; Y axis predicted case numbers.

The Government of Victoria adopted a “stepped” response to their second wave COVID-19 outbreak, from Stage 2 to Stage 3, then to Stage 4, as described in the Supporting Information. This is modelled by the yellow epidemic curve in Fig. 2, which realistically predicts the dynamics of the reduction in daily case numbers up to 30th October 2020, as in Fig. 1.

The rapid increase in diagnosed cases in greater Melbourne from 13th June 2020 onwards (see Fig. 1) suggests that an opportunity existed to move from Stage 2 measures directly to Stage 4. Between 19th June and 29th June case numbers were observed to double every 7 to 10 days, with observed exponential case growth, Fig. 1. Data in Table 1, illustrated in Fig. 2, predict the potential benefit of early activation of Stage 4 “lockdown” measures under alternative activation dates, 19th June, 29th June, 10th July (date of Stage 3 activation), the blue, orange and grey curves respectively, Fig. 2. These illustrate the benefit, in terms of case number reductions, achieved by early activation strategies, compared with the actual date of Stage 4 lockdown on August 3rd, yellow curve Fig. 2.

**Table 1:** Predicted case numbers resulting from earlier Stage 4 activation in greater Melbourne (population 5 million). All scenarios assume schools are closed, and 70% case isolation for adults and 100% for children.

Date	Stage 4, 19th June		Stage 4, 29th June		Stage 4, 9th July		Stage 4, 3rd Aug	
	new	total	new	total	new	total	new	total
16/7/20	232	5884	251	6367	392	8005	455	8623
16/8/20	55	9689	68	10,544	91	13,975	235	20,975
16/9/20	23	10,622	14	11,544	25	15,503	48	24,326
16/10/20	3	10,904	5	11,749	6	15,897	10	24,996
16/11/20	1	10,972	0	11,790	3	16,006	1	25,139
16/12/20	1	10,987	0	11,790	0	16,037	1	25,210
16/1/21	2	10,998			0	16,040	0	25,219
16/2/21	2	11,009					0	25,222
16/3/21	0	11,015						

Table 1 highlights how moving to a Stage 4 “hard lockdown” on 9th July would have reduced total case numbers by approximately 40%, from ~ 25,000 to ~ 16,000 cases, up to May 2021. This corresponds to the smaller area under the grey curve (9th July activation) in Fig. 2 compared to that under the yellow epidemic curve, which predicts total cases resulting from actual Stage 4 activation on 3rd August. Activating Stage 4 measures even earlier, between 19 and 29th June, is shown to be even more effective, reducing total case numbers by over 50%.

Table 1 indicates that under all activation timings, Stage 4 measures are predicted to result in single digit case numbers by the end of October 2020, and effectively halt virus transmission by early 2021. Modelling the impact of the stepped measures introduced by the Government of Victoria, involving Stage 3 measures applied on 9th July and Stage 4 on 3rd August, accurately predicted this decline in case numbers. This is observed by comparing the yellow predicted 3rd August epidemic curve in Fig. 2, with diagnosed case data up to 30th October 2020, Fig. 1.

### Response triggers

Throughout May 2020, daily case numbers in greater Melbourne had been steady and in single figures. As in Fig. 1, reported daily case numbers began to increase throughout June and into July. Data generated by our modelling analyses (Table 1) predicts that Stage 4 lockdown activated on 29th June, when daily case numbers had grown to 61 (see paragraph below), may have reduced total case numbers by over 50%. If activated 10 days later, on 9th July, the reduction in total case numbers is less, reducing numbers by approximately 40%.

The following data sequence is from daily COVID-19 case data published by the Government of Victoria^7^; 5 cases on 11th June, 10 on 13th, 20 on 16th, 25 on 19th, 33 on 24rd, 40 on 26th and 61 on 29th June 2020, 98 on 5th July, 122 on 7th, 143 on 9th and 218 on 14th July^7,8^. Note that reported case data is known to vary day-to-day, as seen in Fig. 1, depending on numbers attending testing facilities, and varying between weekend and working-week days. However, a clear pattern can be seen with this data sequence, with cases doubling approximately every 8–12 days from 16th June to 9th July, giving an exponential increase over that period, then increasing linearly until mid-August 2020. This is illustrated in Fig. 1^8^. The above results suggest that the exponential increase in diagnosed cases during that period could have been used to trigger hard lockdown measures. If used, this triggering approach is predicted to reduce virus transmission and resulting case numbers.

### Activation of stronger social distancing, 9th July 2020

**Figure 3:**
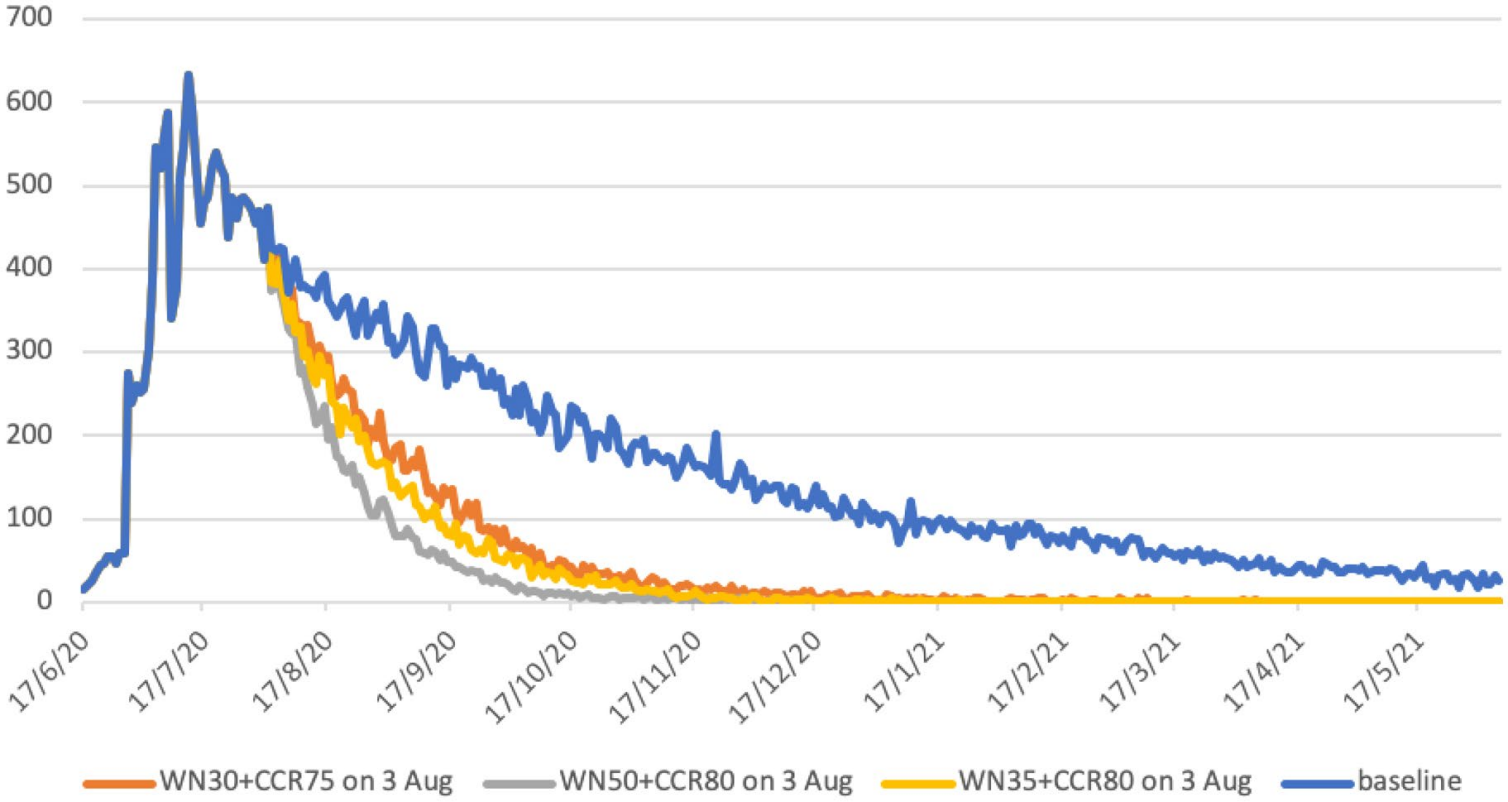
Increased social distancing activated on 3rd August 2020 in greater Melbourne (population 5 million). Grey curve models Stage 4 measures applied. Yellow and orange curves allow greater workplace activity, increasing workplace attendance to 70% and 65% respectively. Blue curve represents continuation of Stage 3 measures, i.e. no increase in social distancing. All scenarios assume schools are closed, and there is 90% case isolation for adults and 100% for children. X axis gives calendar dates; Y axis predicted case numbers.

The State of Victoria responded to the COVID-19 outbreak by increasing social distancing measures in two steps; Stage 3 measures on 9th July 2020, and given the continuing increase in case numbers, strengthened measures to Stage 4 on 3rd August 2020^21^. Modelling was conducted to analyse the impact of this stepwise series of measures, and contrast these with the situation had they not occurred. Stage 3 measures introduced on 10th July 2020, namely (SC100 + WN20 + CCR60 + CI70), see Table S1 Supporting Information, were strengthened to Stage 4 on 3rd August, estimated to increase workplace non-attendance to 50% and reduce community-wide contact to 20%, an 80% reduction. This resulted in social distancing scenario (SC100 + WN50 + CCR80 + CI70), the grey curve in Fig. 3.

Data generated by these modelling experiments (Table 2) highlight the need for, and effectiveness of, the hard lockdown measures taken, grey curve Fig. 3. Without them virus transmission is predicted to continue to at least June 2021 had the Stage 3 measures been maintained indefinitely, blue curve Fig. 3.

**Table 2:** Increased social distancing measures activated on 3rd August 2020, greater Melbourne (population 5 million). Daily cases mid-month and cumulative cases to 16th May 2021. All scenarios have Stage 3 measures activated on 10th July 2020, all schools are closed, and 70% case isolation for adults and 100% for children is assumed.

	Baseline Stage 3 10th July (no Stage 4)		WN50 + CCR80 Stage 4 3rd August		WN30 + CCR75 3rd August		WN35 + CCR80 3rd August	
	Daily	Total	Daily	Total	Daily	Total	Daily	Total
16/08/20	393	22,313	235	20,975	288	21,642	271	21,387
16/09/20	260	32,393	48	24,326	129	27,628	81	26,365
16/10/20	200	39,801	10	24,996	42	29,883	32	27,960
16/11/20	170	45,630	1	25,139	17	30,735	9	28,488
16/12/20	121	49,875	1	25,210	14	31,082	2	28,601
16/01/21	93	53,088	0	25,219	0	31,230	0	28,643
16/02/21	72	55,725	0	25,222	1	31,306	0	28,650
16/03/21	59	57,658			1	31,355		
16/04/21	41	59,161			0	31,382		
16/05/21	29	60,294			0	31,386		

Further modelling experiments were conducted to evaluate whether less restrictive Stage 4 strategies may have had a similar benefit. The aim of these measures would be to lessen the economic impact of businesses closing due to hard lockdown, by increasing workplace attendance.

This was achieved by (a) allowing small service businesses to reopen, i.e. cafes with staff returning, and (b) larger workplaces increasing numbers working in-situ, rather than remotely. We evaluated two scenarios: (1) reducing workplace non-attendance to 30% (so increasing attendance to 70%) and lessening the community contact reduction to 75%, and (2) having workplace non-attendance at 35% and community contact reduction staying at 80%. These gave the orange and yellow epidemic curves in Fig. 3, respectively.

For all three lockdown strategies, these changes in social distancing are predicted to result in minimal virus transmission from mid-January 2021 onwards, with diagnosed case numbers at zero or one for all three strategies (see Table 2, Fig. 3). The strategy reducing case numbers most rapidly corresponds to the Stage 4 measures that were adopted, and described previously (Fig. 2, Table 1).

Strategies with a smaller reduction in workplace non-attendance, to (SC100 + WN30 + CCR75 + CI70) or (SC100 + WN35 + CCR80 + CI70), also result in case numbers decreasing rapidly. Unsurprisingly, the weaker alternative strategy of (SC100 + WN30 + CCR75 + CI70) is less effective in terms of outbreak duration, compared to that with workplace non-attendance at 35% and community contact reduction staying at 80%, see Table 2. The reduction in the total number of cases is approximately linear according to the strength of measures, from 31,386 for the strategy with highest workplace attendance of 70% (WN30), to 28,650 for workplace attendance of 65% (WN35), and 25,222 for the Stage 4 measures used, with workplace attendance at 50% (WN50). This gives reductions in cases of 47.7%, 52.3% and 58% respectively.

Predicted case data in Table 2 indicate that all three increased social distancing strategies are effective in reducing epidemic duration, with daily case numbers in single figures by December 2020 or early 2021. This contrasts to the situation which would result if Stage 3 measures had remained in place, illustrated by the blue case curve in Fig. 3, and case data in the first columns of Table 2. Under that scenario, virus transmission and new cases are predicted to occur up to and beyond May 2021.

## Discussion

Early and robust application of social distancing measures are known to be an appropriate response to the COVID-19 pandemic. In early 2020 such responses were highly effective in minimizing case numbers and the rate of epidemic growth at the early stages of the pandemic, as in Korea and China^22–24^. Similarly, slow responses have resulted in significant first and second pandemic waves, *e.g.* in Italy, Spain and the UK^25^.

This study’s aims were to provide further evidence on the benefit of, and need for, early and robust interventions to contain second waves of coronavirus infections. We quantified the effectiveness of a range of response measures, in terms of the reduction in case numbers, and thus resulting hospitalisations and deaths. These results are intended to help inform the hard COVID-19 containment decisions that need to be made by politicians and public health authorities, until an effective vaccine becomes available. A challenge in responding to COVID-19 second waves is to balance the effect of necessary lockdown measures, in protecting the health of the population, with the negative impact which strict social distancing measures have on a country’s economy. A fundamental issue highlighted in this study is the delay between increasing coronavirus transmission rates, and the time when these manifest themselves as an increase in diagnosed cases. Given that case data always lags the date of virus transmission, our findings indicate that activation of early lockdown is possibly the only feasible strategy to adopt.

Our study was fortunate in having access to comprehensive case data from the rapidly developing COVID-19 second wave in greater Melbourne, Australia from June 2020 onwards. Genomic sequencing has shown that most infections in this second wave result from a breakdown in hotel-based quarantine, followed by two large family gatherings^1,2^. To our knowledge, there were no further introduced infections. This provided a suitable test-bed to analyze the effectiveness of the mitigation measures taken, determine their failings, understand why the second wave spread so rapidly, and to evaluate alternative mitigation strategies as to their effectiveness in reducing the scale of a second wave. These analyses were facilitated by the absence of introduced cases into the modelled population; the lack of “noise” from ongoing infection introduction allowed us to assume that all infections resulted from the single source.

Using second wave outbreak data, we demonstrate how the methodology used realistically modelled SARS-Cov-2 transmission from 1st August to 30th October 2020, when daily diagnosed case numbers had reduced to zero. This prediction of outbreak dynamics provides validation of how lockdown social distancing measures were modelled. That is, the impact which lockdown measures had on reducing transmission over the same period of time. This validation provides evidence as to the robustness of the modelling methods used, and gives credence to the results obtained.

Results indicate that the most effective response, which significantly reduce cases and second wave duration, would be the activation of Stage 4 lockdown measures much earlier, when case numbers were first seen to increase exponentially, on or before 29th June 2020. This approach is estimated to result in ~ 11,000 cases, compared to ~ 25,000 for the strategy taken, in a population of approximately 5 million (Table 2). The second most effective strategy would have been to activate Stage 4 social distancing measures on 9th July, the date of the first response to the second wave, which increased social distancing measures to Stage 3. This is estimated to reduce total cases to ~ 16,000 (Table 2). These two highly effective strategies involved moving directly to Stage 4 measures from Stage 2, rather than the step-wise approach adopted, from Stage 2 to Stage 3 to Stage 4. They highlight the need to “catch” increasing transmission rates before infections are widely distributed, with early *and* robust social distancing contributing to the rapid reduction in virus transmission. The need for timely introduction of lockdown measures is discussed in a short overview of the situation in the UK at the end of October 2020, by Elisabeth Mahase^26^. It appears that the UK has been too slow to introduce hard lockdown, repeating the situation which occurred in greater Melbourne, but on a much larger, country-wide scale. Significantly, the easing of social distancing measures in the UK without high levels of testing and tracing has been shown in a recent modelling study to result in a second COVID-19 wave^27^, predicting the situation which now appears to have occurred^26^.

Had hard lockdown measures introduced on 3rd August in Australia not occurred, we estimate virus transmission would be ongoing beyond the end of our simulation period (mid-May 2021), resulting in approximately 30 cases per day from February onwards (Fig. 3, Table 2). While the Stage 3 social distancing measures activated in early July were predicted to results in a steady decrease in daily cases numbers, they did not lead to virus elimination by mid-2021. As a consequence, this would have limited the Government of Victoria’s ability to ease social distancing and prevent another COVID-19 wave. This would have been a similar situation to that analysed by Di Domenico and colleagues for Northern France^28,29^, and Aleta and colleagues for the North East of the USA^30^.

Our analyses support the decision by the State of Victoria Government to subsequently introduce Stage 4 lockdown measures, which rapidly suppressed ongoing transmission and shortened the duration of the second wave. Once transmission reaches a level where social distancing measures can be safely eased, and new cases managed by highly efficient test, track and isolate systems, this will permit an earlier reopening of businesses and a more rapid increase in economic activity.

There is a challenge in relying on case diagnosis data to trigger activation of strict Stage 4 lockdown measures. Governments are hesitant to introduce such socially disruptive measures given the impact they have on the economy and mental health. However, if the decision to lockdown a city is taken too late, there will have been significant “silent spread” of virus transmission before this increase in infections manifests itself as an increase in diagnosed cases. Rather than using exponential growth in cases as the lockdown trigger, using a threshold of cases per 100,000 of the population is an alternative approach, as is used in the UK. The Australian situation is different in that the goal is to maintain an elimination strategy, via border control and quarantining of international arrivals. A small number of breakout infections into the community from quarantine facilities have occurred in Australia since the large Melbourne outbreak analysed in our study, which itself resulted from such a breakout. These have been successfully controlled by the rapid activation of short-term lockdown measures coupled with high levels of localised testing and contact tracing, as a means of preventing a similar situation to that which occurred in Melbourne in 2020. This early response strategy has allowed lockdown measures to be quickly eased, as soon as daily case numbers are seen to have halted.

We note that the COVID-19 situation in Australia differs from most other countries, and while our key findings are widely applicable, they may not be as effective in other settings due to import of infectious cases. Prior to the second wave there was effectively no community transmission in Australia, due to early and strict border closures. Many countries in Europe, for example, failed to close borders, allowing infectious individuals to spread widely, with resulting widespread transmission, Secondly, the second wave of SARS-Cov-2 transmission was confined to the State of Victoria, particularly to the greater Melbourne area, and ongoing travel restrictions prevented introduction of further infections. This resulted in the effective elimination of virus transmission in Australia by early November 2020^7^.

It should be emphasised the key role which ongoing international border restrictions has had on the COVID-19 situation in Australia. Without the closure of borders to non-residents, together with the use of 14 day hotel quarantining of returning Australian travellers, a strategy of coronavirus elimination in Australia would not have been possible.

Other countries experiencing second waves, or extensions of the first wave, are in a different situation, with limited ability to achieve virus elimination. Other means are needed to reduce transmission levels, and highly efficient testing, contact tracing and household isolation will be needed to contain case numbers to levels which prevent health care facilities from being overwhelmed. Modelling by Aleta and colleagues, in a USA setting^30^, demonstrates how high levels of testing, with 50% of symptomatic cases diagnosed, 14 day quarantine of all members of a case’s household, and precautionary quarantine of contact households, may allow strict lockdown measures to be safely eased. They show that this level of testing and isolation is needed to prevent second COVID-19 waves. Similarly, modelling of methods to ease robust social distancing measures in the Paris region, also indicate that extensive testing and tracing are needed to manage second waves^28,29^. The practical use of this response methodology can be seen with South Korea, which adopted a sophisticated test/trace/isolate approach from the early stages of the pandemic^22,23^. The effectiveness of this approach has been shown to prevent second waves developing^31^.

As with all model-based studies, there are limitations on what features we are able to replicate in detail, and what approximations need to be taken. These involve availability of data, both at the virus transmission level and the population level. We used an estimated basic reproduction number obtained from data gathered in Wuhan, China prior to social distancing activation, and used that to estimate the probability of transmission between two individuals. Detailed census data were used in model development, to create households, workplaces, and education establishments in as much detail as data sources permitted. This allowed us to model movement of individuals between their homes and work and education locations. However, mobility in the wider community was estimated based on the probability of random contact between pairs of individuals, weighted by distance from their homes. Obtaining data on actual population mobility, before and during periods of social distancing restrictions, would aid the fidelity of individual-based models such as ours, but obtaining such data is clearly a challenging task. While others have accessed de-identified mobile phone data to estimate movement throughout a population, as in^30^, an example of how identified data can be accessed and applied in practice is given by South Korea. Here there is general support for government agencies having detailed location and mobility data for the whole population, as a public good. South Korea invested in Information Technology systems to manage future pandemics following the SARS outbreak, and has been highly successful in keeping COVID-19 case numbers low^22,31^. Heavy use of mobile phone data to track the majority of the population, instructing individuals in geo-located “hotspot” areas to be tested, checking on compliance with home isolation, and informing residents of specific areas going into lockdown^22,23,31^.

A further limitation of our study is the use of daily diagnosed case numbers as a surrogate for coronavirus transmission occurring between 5 and 15 days earlier, an approximate propagation time period from date of infection to symptom emergence, time to be tested, and the return of results. Diagnosed case data has a shorter propagation delay than daily hospitalisation and mortality data, and so gives an earlier indication of increasing levels of transmission, and can act as a trigger to increase social distancing measures, as described above. However, this use of case data suffers from the need to make assumptions on the percentage of the population who are asymptomatic following infection, and the percentage of symptomatic individuals who attend testing facilities. In this study we assumed that 35% of those infected were asymptomatic, and that approximately half of the remainder were diagnosed. COVID-19 hospitalisation data may act as a more accurate snapshot of previous rates of transmission, but with a longer propagation delay from time of infection.

The effect of social distancing measures on transmission rates was modelled directly, by reducing person-to-person contact in schools, workplaces and the wider community. These measures assumed that household contact remained. Case isolation was modelled by stopping movement of diagnosed cases out-with the home, allowing for a certain percentage of non-compliance. We assumed 70% compliance by adults and 100% for children, in the absence of published data sources. Availability of contact pattern survey data during the pandemic may have improved the fidelity of our model, and there is a hope that such data will be obtained and available for future pandemic situations.

### Health policy implications

Results from this study reinforce, and furthermore quantify, the benefit of early activation of robust response measures to second (and more) COVID-19 infection waves. Such measures are shown to significantly contain then reduce the epidemic growth rate, and consequential pressure on health care resources. Results demonstrate the criticality of the timing of activation, where a slow response to rapid, exponentially growing case numbers allows the coronavirus to spread widely within the population, before the introduction of more robust social distancing measures can take effect. The study also shows the benefit arising from border closure measures adopted for non-Australians by the Australian Government in late March 2020. These prevented the ongoing introduction of SARS-CoV-2 into the community, until a breakdown in international arrival quarantine measures initiated the second wave of transmission.

**I**n the absence of prospective testing, policy makers rely on diagnosed case, hospitalization and mortality data to inform decision making. All three metrics have inherent (and increasing) time lags; between date of infection and becoming infectious, and the possibility of being diagnosed, hospitalized or dying, with case data having the shortest “propagation delay”.

This study used a discrete COVID-19 second wave in Australia to demonstrate how exponentially increasing diagnosed case numbers, numbers that doubled every 7–10 days, could have better predicted the need for a significantly earlier activation of lockdown measures. Using daily case data as a lockdown “trigger” reinforces the need for a comprehensive and rapid testing program, as described in a related study of lockdown and exit strategies in France^28,32^. As of early November 2020, many countries worldwide still lack highly effective testing and contact tracing systems, so limiting their ability to gain early warnings of a rapid growth in case numbers, and respond as indicated in this study.

A significant challenge for many countries facing second waves of COVID-19 cases is how to balance the negative effect which lockdown measures have on an economy, against the health of the population. It is clear there is a hesitancy by decision makers to introduce robust social distancing measures, as evidenced by the step-by-step approach adopted by the Government of Victoria, Australia, and evaluated in this study. This hesitancy is understandable. Long-duration school closure impacts education outcomes, particularly among those from low socio-economic backgrounds. Closure of cafes, restaurants and bars results in under-employment of young adults, and has a knock-on effect on the economy. Closure of service and transport industries results in increased unemployment, with these measures and home isolation impacting on the mental health of those affected.

Using data from the COVID-19 second wave in Australia, and analyzing alternative response strategies, the study determined the benefit of going hard and early. This contrasts with the approach take in Victoria, where social distancing measures were increased incrementally, resulting in the necessary hard lockdown measures being activated after the coronavirus was widely distributed within the population. This study suggests the optimal response strategy would be to go into lockdown much earlier, resulting in a significant reduction in case numbers, consequential hospitalisations and, potentially, a reduction in the mortality rate.

## Supplementary Information

Supplementary Information.
