# Supplementary material for: A modelling analysis of the effectiveness of second wave COVID-19 response strategies in Australia
Source: Sci Rep. 2021 Jun 7;11:11958. doi: 10.1038/s41598-021-91418-6 (PMC8185067; doi:10.1038/s41598-021-91418-6)
Supplement: Supplementary file 1 — Supplementary Information. [file 41598_2021_91418_MOESM1_ESM.docx]

**Supporting Information**

A Modelling Analysis of the Effectiveness of Second Wave COVID-19 Response Strategies in Australia

George J Milne^1,*^, Simon Xie^1^, Dana Poklepovich^1^, Dan O’Halloran^3^, Matthew Yap^2^, David Whyatt^2^

1. Mathematical Sciences, University of Western Australia
2. School of Medicine, University of Western Australia
3. Department of Health, Queensland Government, Australia

*corresponding author

## Methods

An individual-based model capturing the demographics and movement patterns of individuals within an Australian city, together with SARS-CoV-2 virus transmission data from the early outbreak in Wuhan, China prior to social distancing activation [9], was developed and applied. This was used to analyse the effectiveness of a broad suite of non-pharmaceutical, social distancing interventions, by varying their strength, their time of activation, and their duration. Individual-based (c*.f.* agent-based) modelling is an appropriate methodology to adopt for this task. It permits the effect of four key social distancing measures to be readily captured at a high degree of detail: school closure; reduction in workplace participation; community-contact reduction; and case isolation. This modelling method has been applied previously, to quantify the impact of pandemic mitigation strategies [10-13], and to inform policy decision making [14-17].

This study utilised a model of Newcastle, a city in New South Wales, Australia (population 272,407), whose population demographics reflect Australia as a whole and results were scaled to greater Melbourne, population ~5 million, following an approach used previously [11, 12]. Australian Bureau of Statistics (ABS) census data were used to capture age-specific demographics of every household in the community.[18, 19] ABS workplace data was used to assign adults to workplaces [20], and State Government schools data was used to assign children to age-specific classes [21]. These data were used to model the time-changing contact patterns for each individual, as they move between their household, school/workplace contact hub and in the wider community.

Model parameter settings were calibrated to reflect the transmission characteristics of the COVID-19 epidemic: an incubation period averaging 6 days, from infection to symptom emergence (if any); a latent period averaging 5 days, from infection to infectious; an infectious period averaging 4.5 days, the first day being asymptomatic; and 35% of cases are asymptomatic. The probability of virus transmission from infectious to susceptible individuals was derived from a R_0_ of 2.25, based on SARS-CoV-2 transmission characteristics from Wuhan, China prior to introduction of containment measures [9, 22], following the method applied for pandemic influenza [23].

Model outputs obtained by running the simulation software produced the infection history of every individual in the community, generating the daily (and total) number of infectious individuals, and determining where and when infection occur, as described previously [14, 17]. Modelling analyses were conducted for alternative social distancing strategies, by varying the strength of measures and their activation timing. This quantified how alternative mitigation strategies may have performed, allowing us to contrast alternative mitigation strategies with those that were used. The difference in total case numbers provides a measure of the effectiveness of alternative mitigation strategies to reduce the impact of the outbreak.

Our model depends on a small number of stochastic parameters, including the probability of virus transmission between an infectious individual and a susceptible individual, and the random seeding of infectious individuals into the community to initiate an outbreak. This results in variation between successive simulation runs. From experience with prior, related analyses of mitigation strategy effectiveness for pandemic and seasonal influenza [14, 15, 23], averaging infection data generated from multiple runs stabilises after approximately 16 runs. The results presented here were obtained from multiple simulation runs for each social distancing scenario evaluated.

## Social distancing

Four social distancing measures are available to health authorities, and were combined during the Melbourne COVID-19 outbreak. School closure (SC): reduction in school attendance. Workplace non-attendance (WN): a percentage of all persons in the workforce remain at home during working hours. Community contact reduction (CCR): contact in the wider community is reduced by a given percentage to reflect strength of intervention. Case isolation (CI): a percentage of adults and all children withdraw to the home on becoming symptomatic.

Stage 2, Stage 3 and Stage 4 measures applied in greater Melbourne are described in Table 1 and the Supporting Information [3-5, 24, 25]. These have increasing strength, with Stage 4 lockdown restricting individuals to their homes unless they have approved occupations, require healthcare, or require to shop for essential supplies. The sequence of changes to social distancing measures in the State of Victoria are as follows: Stage 2 measures were activated on 25^th^ March, and given absence of community-wide transmission started to be eased on 26^th^ May, with some students returning to schools. From 26^th^ June all schools closed, mirroring Stage 2 measures. Stage 3 measures were introduced from 2^nd^ July onwards, first to limited areas of greater Melbourne, designated by their postal codes. Stage 3 measures were then extended to all of greater Melbourne on 10^th^ July, however schools reopened on 13^th^ July for years 11 and 12, and for all ages on 20^th^ July. As daily case numbers continued to increase over that period, Stage 4 lockdown measures were activated on 3rd August.

Table S1: *Social distancing measures applied in greater Melbourne [3-5, 24, 25]*. *WN: percentage workplace non-attendance; SC: percentage reduction in school attendance; CCR: percentage reduction in community-wide contact; CI: percentage adult case isolation, all child cases up to and including age 17 are assumed to isolate.*

| Date measures implemented | Social distancing measures | Estimated social distancing |
| --- | --- | --- |
| 25^th^ March | **Stage 2** restrictions commence |  |
| 26^th^ May | Pre-primary, years 1, 2, 11, 12 students return to school, after holiday period. | WN20  SC30 CCR20  CI70 |
| 9^th^ June | Years 3 to 10 back in school. | WN20  SC0  CCR20  CI70 |
| 26^th^ June | School holidays start (as planned). | WN20  SC100  CCR20  CI70 |
| 2^nd^ July | **Stage 3** Stay-at-Home restrictions in ten Melbourne postcode areas. | WN20  SC100 CCR60  CI70 |
| 9^th^ July | **Stage 3** Stay-at-Home restrictions extended to all of greater Melbourne. | WN20  SC100  CCR60  CI70 |
| 20^th^ July | All ages return to school. | WN20  SC0  CCR60  CI70 |
| 3^rd^ August | **Stage 4** restrictions applied to greater Melbourne, including 8pm to 5am curfew.  All schools closed. | WN50  SC100  CCR80  CI70 |

The effect of Stage 3 and Stage 4 social distancing measures on person-to-person contact patterns was estimated from the following government directives, such as for age-specific school non-attendance. Workplace and community-wide contact reductions were estimated from the directives below, and from observation of commuter traffic reductions. The case isolation setting allowed for a limited level of non-compliance.

Stage 3 restrictions (9^th^ July 2020)

Stay at home restrictions, unless essential work, care-giving, shopping for essentials, medical needs.

No travel from regional Victoria into metropolitan Melbourne.

Restaurants and cafes takeaway only.

Libraries and community venues closed except for hosting weddings, funerals, school use.

Weddings limited to 5 people, funerals limited to 10 people.

Cinemas, zoos, wildlife parks, galleries, museums, concert venues, campgrounds, caravan parks closed.

Non-essential retail closed. Essential retail includes food/supermarkets, fuel, hairdressers etc.

Stage 4 restrictions, in addition to those for Stage 3 (3^rd^ August 2020)

Schools closed

Mandatory face masks outside home.

Curfew in place from 8pm to 5am (must be at home, except for essential work, medical care, and caregiving).

No visitors to home.

Non-essential travel limited to 5km from home.

Outdoor exercise limited to 1x 1hr session

Only one person per household can leave home for necessary goods and services

## Model calibration

The “second wave” Melbourne COVID-19 outbreak originated in late May 2020, as a result of a breakdown in hotel quarantine [1]. The scale of the outbreak became apparent later, as diagnosed case numbers started to increase rapidly. A calibration process was used to estimate the initial phase of the outbreak, to create a sufficient pool of infectious individuals that later transition into diagnosed cases, and thus align with daily reported case numbers. The calibration aligned the daily number of new infectious individuals in the population generated by the simulation model, to actual, reported case numbers and used the infection timeline parameters given in Supporting Information, Table S1. This allowed us to predict case number dynamics into the future, and under alternative social distancing measures.

An experimental process was used to calibrate actual daily diagnosed case numbers with SARS-Cov-2 infection events in the model. Infection events model virus transmission, when a susceptible individual becomes infected due to contact of an infectious individual on a given day. This was achieved by running the simulation model repeatedly over the first month of the outbreak, with alternative numbers of infectious individuals seeded into the modelled community to initiate the outbreak in the model. The aim was to replicate the early stages of the outbreak as accurately as possible. These simulations resulted in increasing numbers of new infections each day, which were translated into predicted new daily cases occurring at a later point in time, using the infection parameter data in Table S1. By adjusting seeding of infectious individuals into the model in early June 2020, and under the social distancing measures then in place, we were able to select the seeding which best matched the actual daily changes in case numbers appearing at a later time.

The calibration process was conducted in two steps, to reflect an increased testing regime beginning in early July. The first calibration phase resulted in approximately a third of all infected individuals being diagnosed, the remaining two thirds being in either the latent period, or asymptomatic or exhibiting mild symptoms, and thus not accessing a testing facility. In the second phase, when a more aggressive testing regime involving increased contact tracing was initiated from 2^nd^ July 2020 onwards, we assumed a steady increase in the (positive case) diagnosis rate, as the overall testing coverage increased. This ratio, between infections and the percentage of infections being diagnosed and reported as cases, was used to convert daily infection data generated by the model into predicted case numbers.

It should be noted that aligning the occurrence of new infections within a model with actual diagnosed case data is inherently difficult. There is inherent variability in daily case data depending on how the population actually gets tested. Contact tracing and “forcing” individuals to be tested is likely to break down as infection numbers increase; asymptomatic cases are likely to go untested; and the time between infection in an individual occurring and that individual being diagnosed (if at all) will vary substantially across a time window. Thus predicting prior infection patterns from a series of daily testing data will be inherently inexact.

## Strengthening social distancing

Simulation experiments were conducted by running model software, after adjusting the strength of social distancing measures to reflect introduction of Stage3 and Stage 4 restrictions. Random seeding of infectious individuals into the model was used to capture the effect of localized high transmission events, resulting from a number of large gatherings in late June 2020 [2]. The following were determined to replicate daily outbreak case dynamics up to 1^st^ August 2020.

Prior to increases in social distancing measures on 9^th^ July 2020, we assumed 30% of school-age children were not attending school (SC30) and workplace attendance and community contact were both reduced by 20% (WN20) and (CCR20) respectively, as in Table 1. This weak Stage 2 social distancing arose from previous easing of measures, given low levels of virus transmission throughout Australia. Simulations were run from 17^th^ June 2020 onwards with approximately 10 infections seeded daily, and with social distancing at (SC30+WN20+CCR20), as above. From 28^th^ June an additional 180 infected individual were seeded daily to model the rapid growth in infections due to large-scale family gatherings [1, 2].

Following a significant increase in diagnosed cases in late June 2020, the Victorian Government announced Stage 3 restrictions to apply from 9^th^ July 2020. All schools, cafes, restaurants and bars closed, and public gatherings and sporting events stopped, estimated to give a 60% community-wide contact reduction (CCR60) coupled with 100% school closure (SC100), as detailed in Table 1. Case isolation at home was assumed to have compliance of 70% for adults and 100% for children (CI70). The 20% workplace reduction continued, resulting in a (SC100+WN20+CCR60+CI70) Stage 3 social distancing strategy.

Seeding of infectious individuals into the model was stopped on 9^th^ July 2020, thus all further infections in the model occurred as a consequence of the breakdown in hotel quarantine measures and, subsequent large family gatherings. This replicates what is known to have occurred, with genomic sequencing recently showing that over 90% of all outbreak cases were due to a breakdown in quarantine measures, and arose from transmission between infected travellers and quarantine facility staff within a quarantine hotel [1, 2].

Calibrating our model to case data up to the beginning of August, and continuing simulations into 2021, allowed us to analyse the effectiveness of a range of alternative social distancing strategies, and to compare these with those applied by the Government of Victoria.

## Early activation of Stage 3 measures

Additional modelling analyses were conducted to evaluate outcomes from earlier activation of Stage 3 measures. These had the aim of determining whether this change may have reduced the need for introduction of the more robust Stage 4 lockdown measures, and consequential negative economic impact. Figure S1 illustrate the impact of earlier activation of the Stage 3 social distancing measures, from (SC100+WN20+CCR60+CI70) to (SC100 +WN20+CCR60+CI70), five and ten days earlier that the actual 10^th^ July activation date.

*Figure S1: Greater Melbourne (population 5 million) COVID-19 outbreak with earlier Stage 3 social distancing activation. Blue curve, 9^th^ July 2020 actual date of activation; orange curve, earlier activation 4^th^ July; grey curve earlier activation on 29^th^ June. X axis gives calendar dates; Y axis predicted case numbers.*


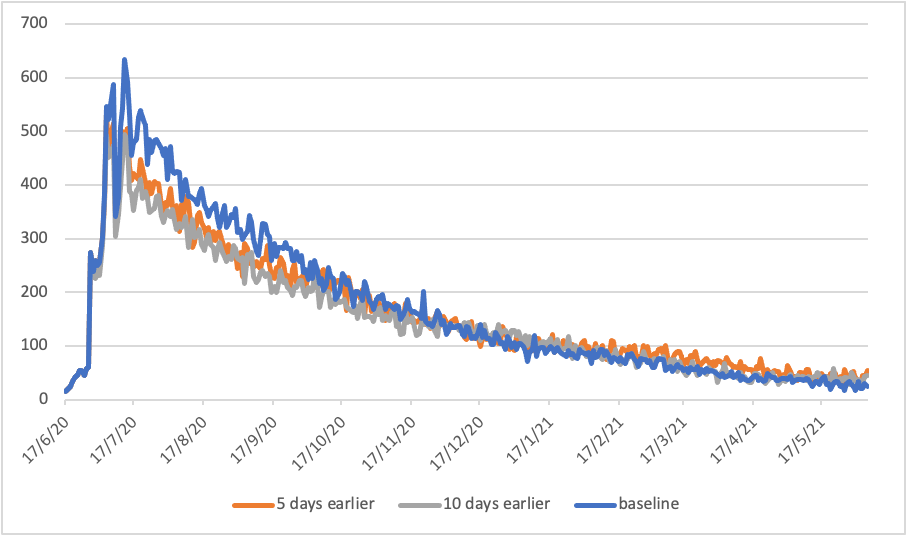


Stage 3 activation on 29^th^ June, 10 days earlier than the 10^th^ July activation, are estimated to result in daily case numbers by mid-September reducing from 260 to 199. Total case numbers to mid-May 2021 are estimated to reduce from 60,294 to 52,475, Table S2. If activated 5 days earlier, on 4^th^ July, when daily case numbers were 64 [7], daily case numbers by mid-September drop from 260 to 233, and the total number up to mid-May 2021 will reduce to 56,920, Table S2.

*Table S2: Daily and cumulative cases to 16^th^ May 2021 for greater Melbourne, population 5 million; Stage 3 social distancing activated on 9^th^ July (baseline), 4^th^ July and 29^th^ June 2020*

| Date | Stage 3 on 9^th^ July | | Stage 3 on 4^th^ July | | Stage 3 on 29^th^ June | |
| --- | --- | --- | --- | --- | --- | --- |
|  | New cases | Total | New cases | Total | New cases | Total |
| 16/7/2020 | 455 | 8,623 | 409 | 7,937 | 385 | 7,444 |
| 16/8/2020 | 393 | 22,313 | 331 | 19,430 | 289 | 18,128 |
| 16/9/2020 | 260 | 32,393 | 233 | 27,977 | 199 | 26,176 |
| 16/10/2020 | 200 | 39,801 | 222 | 34,852 | 181 | 32,279 |
| 16/11/2020 | 170 | 45,630 | 165 | 40,372 | 149 | 37,191 |
| 16/12/2020 | 121 | 49,875 | 120 | 44,599 | 139 | 41,278 |
| 16/1/2021 | 93 | 53,088 | 105 | 47,954 | 108 | 44,929 |
| 16/2/2021 | 72 | 55,725 | 82 | 50,911 | 71 | 47,724 |
| 16/3/2021 | 59 | 57,658 | 81 | 53,346 | 62 | 49,733 |
| 16/4/2021 | 41 | 59,161 | 56 | 55,447 | 32 | 51,243 |
| 16/5/2021 | 29 | 60,294 | 44 | 56,920 | 45 | 52,475 |

Table S2 data and the epidemic curves in Figure S1 suggest that the 10 day earlier activation of Stage 3 measures on 29^th^ June has limited effect on both daily and overall case numbers, up to 17^th^ May 2021. The 5 day earlier activation of Stage 3 measures is predicted to reduce total case number by approximately 10%, while the 10 day earlier activation is predicted to reduce total case numbers by almost 20%.

These data indicate that the slightly earlier activation of Stage 3 social distancing measures fails to significantly reduce the duration of virus transmission, with transmission continuing to at least June 2021. This supports the decision by the Government of Victoria to then strengthen measures to Stage 4 lockdown, with our modelling estimating cessation of transmission by the end of 2020.

## Model parameters

*Table S3: Summary of* SARS-CoV-2  *parameters*

| Name | Value | Source |
| --- | --- | --- |
| Infection Timeline Settings | | |
| Incubation Period | 6 days | [9, 36] |
| Latent Period | 5 days | Estimated |
| Infectious generation period | 4.5 days | [37] |
| Symptomatic period | 3.5 days | [9] |
| Post-symptomatic infectious period | None |  |
|  | | |
| Asymptomatic settings | | |
| Asymptomatic ratio | 35% | [38] |
| Asymptomatic transmissibility | 55% | [37] |
|  | | |
| Calibration Settings | | |
| Strain | 1 | [39] |
| Pre-existing immunity and vaccination | None | [40] |
| R_0_ | 2.25 | [9, 22] |
| Total infections after 360 days | 68.74% | Simulation Output |
